# Assessment of the periodontal health status and gingival recession during orthodontic treatment with clear aligners and fixed appliances: A systematic review and meta-analysis

**DOI:** 10.4317/medoral.25760

**Published:** 2023-01-15

**Authors:** Marina Crego-Ruiz, Adrià Jorba-García

**Affiliations:** 1 DDS. Fellow of the Master’s degree programme in Orthodontics. Faculty of Medicine and Health Sciences, University of Barcelona, Barcelona, Spain; 2 DDS, MS. Master’s degree in Oral Surgery and Implantology, Faculty of Medicine and Health Sciences, University of Barcelona, Barcelona, Spain

## Abstract

**Background:**

The aim was to assess periodontal health maintenance and gingival recessions development in patients undergoing an orthodontic treatment with clear aligners (CA) and fixed appliances (FA).

**Material and Methods:**

An electronic search in MEDLINE, Scopus, The Cochrane Library, and Web of Science was performed up to September 2022 to identify all potential articles. Two investigators independently selected the studies according to the inclusion criteria. Prospective and retrospective studies assessing the periodontal health status and gingival recession development during the orthodontic treatment with buccal FA and CA were included. Case series, cross-sectional studies, and studies with less than two months of follow-up were excluded. Two investigators independently extracted the data from included articles and assessed risk of bias across studies using the Cochrane Collaboration tool. Qualitative and quantitative analyses of the data were performed. Pairwise meta-analysis using a random-effects model were used to compare periodontal indices between FA and CA treatment in different follow-up periods.

**Results:**

From the 129 potential studies, finally 12 studies were included. Only 8 could be included in the quantitative analysis. CA seems to slightly maintain better periodontal health indices. Only plaque index in a mid-term follow-up (mean difference (MD): -0.99; 95%; Confidence interval (CI) [-1.94 to -0.03]; *P*=.04; I2=99%), and pocket probing depth at a long-term follow-up (MD: -0.93mm; 95% CI [-1.16 to 0.7]; *P*<0.0001) reported statistically significant results favoring CA.

**Conclusions:**

Up to the date there is not enough evidence to conclude that CA maintains better periodontal health during an orthodontic treatment than FA.

** Key words:**Clear aligners, removable orthodontic appliances, fixed orthodontic appliances, periodontal health, gingival recession.

## Introduction

The periodontal health maintenance during an orthodontic treatment depends on several factors, such as the host resistance; systemic disease or conditions such as diabetes mellitus or smoking habit; periodontal phenotype, especially regarding the width of the buccal bone plate; the amount and composition of dental plaque; and the oral hygiene habit of the patient - the latter being probably the most critical factor (1,2).

Several studies showed that orthodontic fixed appliances (FA) significantly enhance the accumulation of dental plaque due to the difficulty of maintaining proper oral hygiene, which favors the progressive demineralization of enamel and gingival inflammation which could lead to the destruction of the tooth supporting tissues (3,4). Hence, patients must be instructed to carefully brush and floss around braces and wires to remove all traces of plaque, and clinicians should consider introducing them in a special periodontal maintenance program (5).

Contemporary, removable transparent plastic clear aligners (CA) were introduced to overcome some limitations of FA. The current literature describes it as a safe, comforTable and aesthetic treatment (6,7). Additionally, CA can be easily removed allowing patients to preserve oral hygiene to optimal levels (1,6). Recently, studies assessing periodontal health status in patients undergoing orthodontic treatment with FA and CA had been carried out (8-10). Many clinicians believe that CA are more prone to maintain the periodontal health rather than traditional FA, nevertheless there is not enough evidence to confirm this hypothesis (1,7,11).

Furthermore, the relationship between gingival recessions and orthodontic treatment is under controversy. The prevalence spans 5% to 12% at the end of treatment but could be increased up to 47% in long-term observations (12). Some authors suggest that orthodontic therapy could be associated with alveolar bone loss, gingival recession and loss of clinical attachment level (13). Nowadays, the movement of teeth outside the alveolar ridge bone has been described as risk factor for gingival recession (12,14,15).

To the best of our knowledge, there are already some published reviews addressing the relationship between periodontal health and orthodontic treatment with FA and CA, but some limitations arise from those reviews. In 2015, Rossini *et al*. (1) published a systematic review but could not perform a quantitative analysis of data due to the limited and heterogenous studies published at that time. More recently, a meta-analysis aimed address this topic, but it has some important limitations, such as vague searching strategery and inclusion criteria and improperly pooled results, in fact, they include in the meta-analysis studies that did not use clear aligners (3,16). Additionally, at the present any review aimed to compare the development of gingival recession in patients under orthodontic treatment with FA and CA. Therefore, authors consider that it is justified to update these systematic reviews and meta-analysis to overcome these limitations.

Hence, due to the high demand of orthodontic treatment among patients and the importance of maintaining periodontal health, this systematic review and meta-analysis aims to assess periodontal health maintenance and gingival recessions development in patients undergoing an orthodontic treatment with CA and FA.

## Material and Methods

The present systematic review and meta-analysis adheres to the preferred reporting items for systematic reviews and meta-analyses: the PRISMA Statement (17) and the protocol was registered in advance in PROSPERO under the number CRD42020175280.

The PICOS question of the review was:

1) Population: Patients undergoing orthodontic treatment.

2) Intervention: Orthodontic treatment with clear aligners.

3) Comparison: Orthodontic treatment with fixed appliances.

4) Outcome: Periodontal health status (plaque index, pocket probing depth, gingival index and bleeding on probing) and gingival recession development.

5) Studies: Randomized clinical trials, controlled clinical trials and prospective or retrospective cohort studies.

- Eligibility criteria

The inclusion criteria included randomized clinical trials, controlled clinical trials and prospective or retrospective cohort studies in which the periodontal health status and gingival recession development were assessed and compared in an objective manner during the orthodontic treatment with buccal FA and CA.

Cross-sectional studies, studies with less than 2 months follow-up, studies without a control group and case series or case reports were excluded. Studies assessing lingual appliances, orthodontic retainers or orthognathic surgery were also excluded.

The intervention for all studies was the orthodontic treatment with CA, and the orthodontic treatment with buccal FA was considered as control. Primary outcome was the periodontal health status measured as follows: plaque index (PI), pocket probing depth (PPD), gingival index (GI) and bleeding on probing (BoP). The secondary outcome was the development or increase of gingival recession measured as the apical shift of the gingival margin between the position prior to the orthodontic treatment and the post-treatment position.

- Information sources and literature search

An electronic search in MEDLINE (PubMed), Scopus (Elsevier), The Cochrane Library, and Web Of Science was performed up to 30th September 2022 to identify all relevant articles assessing periodontal health in patients undergoing orthodontic treatment. The search strategy used is summarized in Table 1.

**Table 1:**
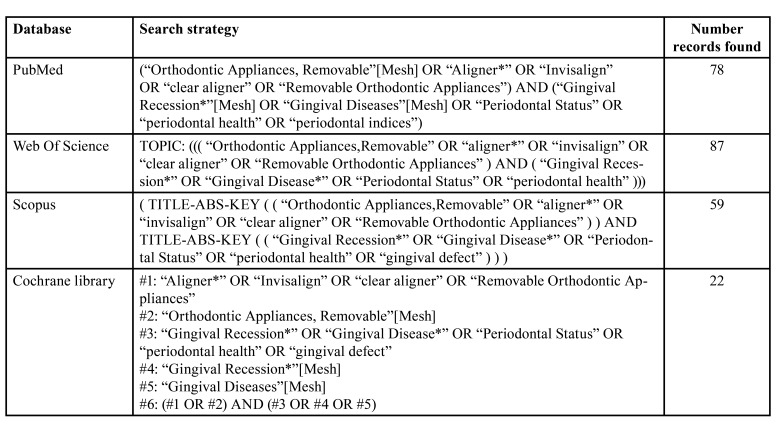
Search strategy.

The search was completed by manual screening of the references in the selected articles. Additionally, ClinicalTrials.gov was searched to detect relevant unpublished data and OpenGrey and www.greylit.org were searched for grey literature.

- Study selection

Two investigators (M.C-R and A.J-G) with prior experience in systematic reviews and meta-analysis independently select the studies according to the inclusion criteria. Any disagreement was solved by consensus, in case no consensus was reached a third independent investigator not involved in the review decided on it.

First, duplicates were removed. Then, irrelevant articles based on title and abstract were excluded. Finally, the full text of the non-excluded articles were assessed for eligibility. Studies removed at this stage were recorded with the reason for its exclusion. A Cohen's kappa coefficient of 0.914 showed a high degree of agreement between the investigators.

When multiple reports on the same sample were identified, the most recent publication was included.

- Data collection and extraction

Two investigators (M.C-R and A.J-G) independently extract the data using data-extraction Tables. The Table includes: author and year of publication, country, type of study, aim, participants’ characteristics, intervention and control, outcomes measured, follow-up, summary of results and reported conflict of interest.

For primary outcomes, the PI, GI, BoP and PPD were recorded at each timepoint of follow-up. For the secondary outcome, the gingival recession was also retrieved from each study.

- Risk of bias assessment

Two reviewers (M.C-R and A.J-G) independently assessed risk of bias (RoB) of the included studies as part of the data extraction process.

For non-randomized studies, the risk of bias in non-randomized studies of interventions (ROBINS-I) assessment tool suggested in the Cochrane Handbook for Systematic Reviews of Interventions (version 6.1) was employed (18). The following items were evaluated: 1) bias due to confounding, 2) bias due to selection of participants, 3) bias in classification of interventions, 4) bias due to deviations from intended interventions, 5) bias due to missing data, 6) bias in measurement of outcomes, and 7) bias in selection of the reported results. Each domain could be judged as low risk, moderate risk, serious risk, critical risk or no information. Finally, an overall risk of bias was assigned to each study: a study could be judged as low risk of bias only if all domains were judged as low risk; if at least one domain has been judged as moderate risk, the study must be considered at least of having a moderate risk of bias. Finally, if a study has at least one domain judged as serious or critical risk, the study must be considering as being of serious or critical risk of bias respectively.

For the included randomized clinical trials, the risk of bias was assessed using the Cochrane Collaboration risk of bias tool (RoB 2.0) suggested in the Cochrane Handbook for Systematic Reviews of Interventions (version 6.1) (19). The following items were evaluated: 1) randomization process, 2) deviations from intended interventions, 3) missing outcome data, 4) measurement of the outcome, and 5) selection of the reported result. Each domain could be judged as low risk of bias, some concern or high risk of bias, then an overall risk of bias for each study was assigned. Studies considered as low risk of bias need to have the 5 domains judged as low risk, if at least one domain has some concern, the overall study must be considered at least as having some concern. If the study has some concern in more than one domain or one domain judged as high risk of bias, the study was considered as being of high risk of bias.

Authors were contacted for clarification of any missing or unclear information when necessary.

- Summary measures and synthesis analysis

A descriptive analysis of the included articles was performed in a descriptive summary: 1) author, 2) year, 3) country, 4) study design, 5) number of participants, 6) age, 7) gender, 8) intervention, 9) comparison, 10) periodontal indices measured, 11) follow-up, 12) professional oral health program, and 13) conflict of interest.

If studies reported the index values in the baseline and for each time point of the follow-up but do not report the change, the means of the follow-up and baseline were subtracted in order to obtain the change from the baseline. Standard deviation was imputed as recommended in the Cochrane Handbook for Systematic Reviews of Interventions (version 6.1). Additionally, although GI and PI are ordinal variables, they were considered as continuous variables as proposed in the Cochrane Handbook for Systematic Reviews of Interventions (version 6.1).

Pairwise meta-analyses were used to compare the outcomes assessed in the group FA and CA. Meta-analyses were only performed when studies reported the same periodontal indices at the same follow-up. Mean differences were combined using random-effects models, which was considered more appropriate due to the heterogeneity between studies regarding settings and population.

For each periodontal index, three meta-analyses were performed to assess each index in different follow-up time points. We grouped data in: short-term (from baseline to 2-3 months), mid-term (from baseline to 6-9 months) and long-term ( from baseline to 12 months or more).

Heterogeneity was explored with the I2 analysis and the χ2 (Q value). An I2 value of >50% were interpreted as significant heterogeneity. If high heterogeneity was detected and sufficient studies were included in the metanalysis, studies were isolated in subgroups and subjected to linear meta-regression to identify them as possible sources of covariance. If the meta-analysis included less than 10 studies, neither heterogeneity with sensibility tests nor publication bias was explored.

Statistical analysis was carried out with Stata 14 software (StataCorp, College Station, TX, USA), and the software Review Manager (version 5.3) (The Cochrane Collaboration, Copenhagen, Denmark). The level of significance was set at *P* < 0.05 for all analyses.

## Results

- Study selection and characteristics

**Figure 1:**
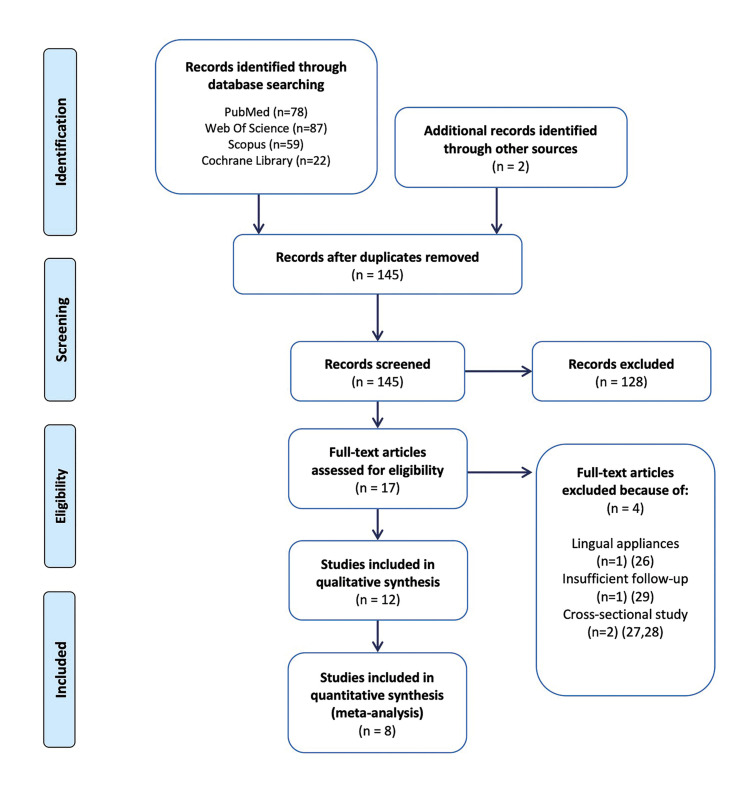
Flow diagram outlining the articles selection for the systematic review according to the PRISMA guidelines.

From the initial 145 potential articles, finally only 12 (4,5,8-11,20-25) studies met the eligibility criteria. Four articles were excluded after the full text evaluation (26-29), reasons are reported in Fig. 1.

Three studies were randomized clinical trials (4,5,10), eight prospective cohort studies (8,11,20-25) and one retrospective cohort study (9). From these studies, eight studies (4,5,8-10,21-23) were introduced into the quantitative analyses while the other three (11,20,24,25) were excluded due to the use of different periodontal indices or insufficient reported data. Flow diagram with the selection study process and reasons why excluding studies in the final full-text screening stage are reported in Fig. 1.

Included studies involve a total of 612 patients; 291 treated with CA and 321 with buccal FA. Regarding periodontal health status indices, the most analyzed indices were PI, GI, PPD, and BoP. Most of the studies include young patients between 15 and 30 years old, but Han (23) included older patients with a mean age of 52.9 ± 9.42 years old and a background of periodontal disease. All the studies promote oral health at least once during the orthodontic treatment, most of them performed a professional oral hygiene and reinforced oral health instructions before starting orthodontic treatment (5,8,9,19,22,24,25). Additionally, other studies reinforced oral health instructions periodically during the treatment (4,8-10,21). The description of included studies is summarized in Table 2.

- Risk of bias within studies

The RoB assessment is summarized in Table 3 and 4. Only one study was rated positively in all the domains and could be considered at low risk of bias (4). The main limitation that arises in randomized and non-randomized studies is the blinding of the outcome assessor (5,8-11,20-25). This issue is difficult to addressed given that FA are not removed in orthodontic follow-up visits. The majority of the randomized clinical trials reported high risk of bias arising from the randomization process (5,10). Some non-randomized studies received a moderate risk of bias due to the selection of participants since some studies started time after the assignment of the exposure (8,9,21,22). Interestingly, the age between groups was markedly different in two studies (8,9) and this could be an important source of bias since the oral hygiene habit could be different between teenagers (16-20 years old) and adults (30-40 years old).

**Table 2:**
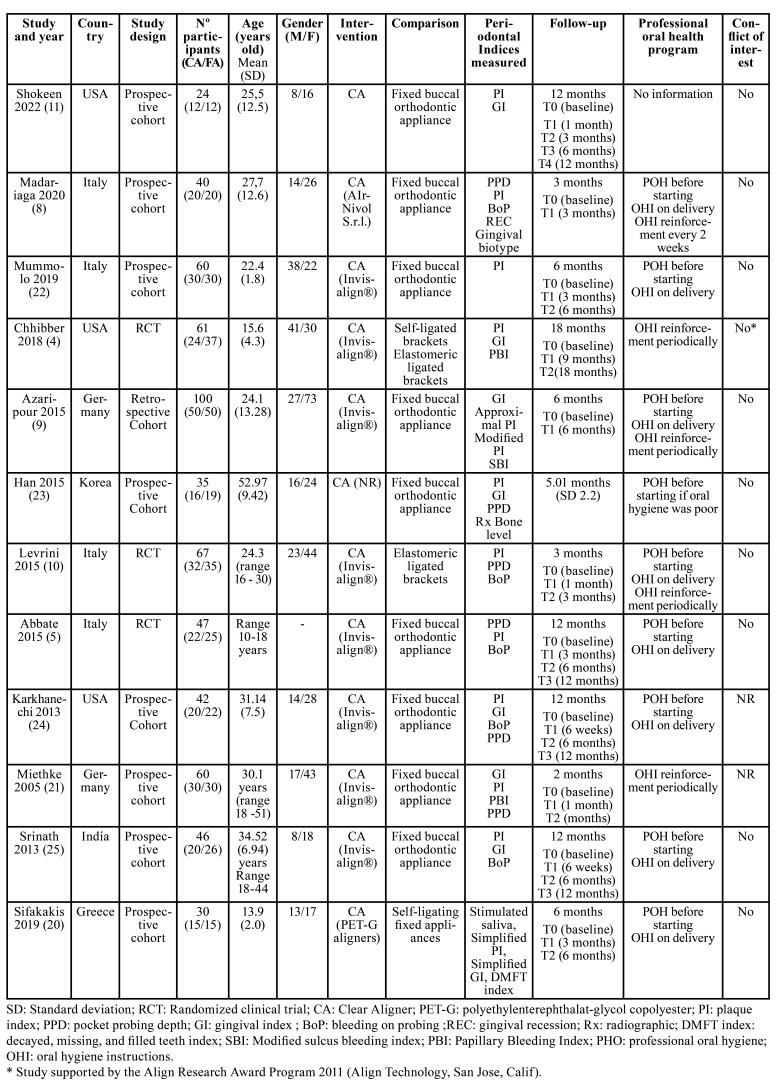
Description summary of the selected studies.

**Table 3:**
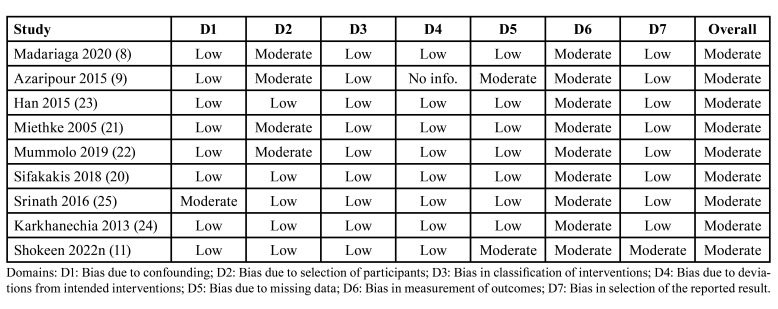
Summary of the risk of bias of the selected non-randomized trials.

**Table 4:**
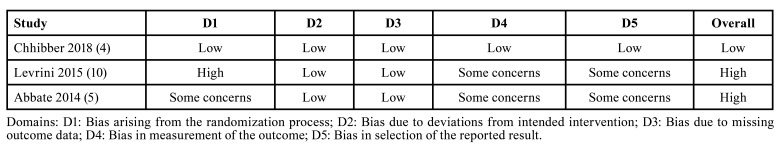
Summary of the risk of bias of the selected randomized clinical trials.

- Results of individual studies, meta-analysis, and additional analyses

Plaque index (PI)

**Figure 2:**
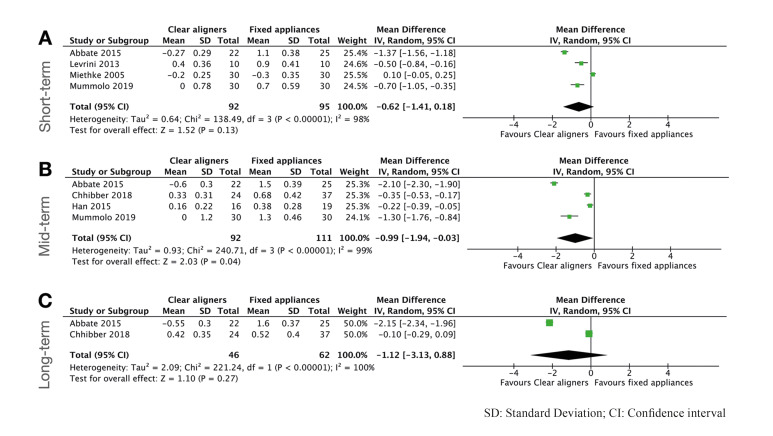
Forest plots for Plaque index comparing clear aligners versus fixed appliances. A) short-term follow-up; B) mid-term follow-up; C) long-term follow up.

PI was assessed in 6 studies included in the quantitative analysis (4,5,10,21-23). The quantitative analysis of PI yielded significantly better results favors to CA in a mid-term follow-up (mean difference (MD): -0.99; 95%; Confidence interval (CI) [-1.94 to -0.03]; *P*=.04; I2=99%). Regarding short and long-term follow-up, no statistically significant differences were found between both modalities although there was a tendence of reporting better PI scores in the CA group. Forest plots can be observed in Fig. 2. The four studies (11,20,24,25) that could not be included in the meta-analysis had also better results of PI for CA, and the PI increases during the follow-up compared to the baseline specially at 6 and 12 months follow-up.

Gingival index (GI)

**Figure 3:**
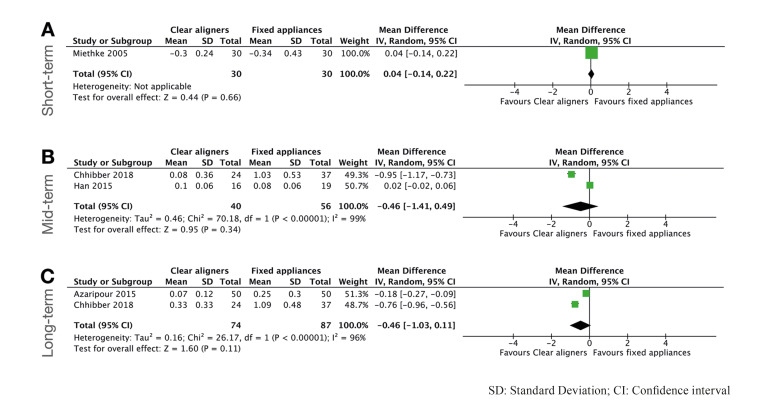
Forest plots for Gingival Index comparing clear aligners versus fixed appliances. A) short-term follow-up; B) mid-term follow-up; C) long-term follow up.

This index was reported by few studies, thus only 4 studies could be included in the meta-analysis (4,9,21,23). According to the weighted mean differences, no statistically significant differences were detected for any term follow-up (*P*>0.05). Interestingly there was also a tendence of reporting better index values for CA, particularly in a long-term follow up (MD: -0.46; 95% CI [-1.03 to 0.11]; *P*=0.11; I2=96%) (Fig. 3). Regarding the studies not included in the quantitative analysis (11,20,24,25), similar results were found, and statistically lower GI scores were reported in CA than in FA group.

Pocket probing depth (PPD)

**Figure 4:**
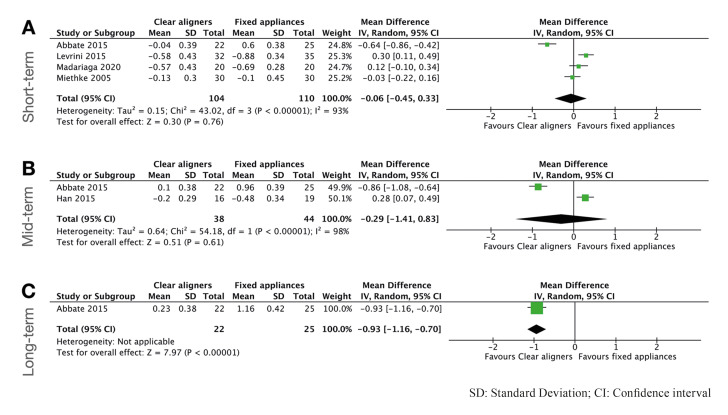
Forest plots for Pocket probing depth comparing clear aligners versus fixed appliances. A) short-term follow-up; B) mid-term follow-up; C) long-term follow up.

Concerning PPD, five studies were included in the quantitative analysis (5,8,10,21,23). No statistically significant differences were found between both approaches in a short and mid-term follow up (*P*>0.05). Nevertheless, in a long-term follow-up, only one study reported data regarding this outcome and the results for the meta-analysis yielded statistically significant results favoring CA (MD: -0.93mm; 95% CI [-1.16 to 0.7]; *P*<0.0001). Forest plots summarizing the results can be observed in Fig. 4.

Bleeding on probing (BoP)

BoP was assessed in 8 studies (4,5,8-10,21,24,25) whereas different measuring indices were employed, hence no meta-analysis could be performed. All studies but Miethke and Vogt (21) reported statistically significant better indices of bleeding in the CA treatment group than in FA. Interestingly, Karkhanechi *et al*. (24) and Srinath *et al*. (25) did not find differences at short and mid-term follow-up but they did at long-term (12 months) follow-up. On the other hand, Chhibber *et al*. (4) found statistically significant differences at 9 months follow-up but did not find it at 18 months follow-up.

Gingival recession

Finally, regarding gingival recessions, only one recently published study reported this outcome. Madariaga *et al*. (8) found that patients wearing FA had statistically significant more gingival recession at the follow-up visit (after 3 months) compared to the baseline: -0.85mm ± 0.45 and -0.67mm ± 0.51 respectively. On the other hand, no differences between the baseline and the follow-up regarding the gingival margin position were found in the CA treatment group; the mean gingival recession was -1.16mm ± 0.18 at both time points.

- Risk of bias across studies

Analysis of publication bias was not undertaken as no more than 4 studies were included in an individual meta-analysis.

## Discussion

The present review evaluated periodontal health maintenance during an orthodontic treatment with FA and CA. Despite the limited data, periodontal indices demonstrate a slight tendence of having better periodontal health status in patients treated with CA. Nevertheless, meta-analysis only reported statistically significant results when assessing PI in a mid-term follow-up and PPD in a long-term follow up, hence, up to the date there is not enough evidence to accept the hypothesis that CA maintains better periodontal health during an orthodontic treatment, and it seems that orthodontic appliances itselves have a little to null effect on the periodontal health.

Even though the results reported in the quantitative analysis were statistically significant, its clinical relevance could be limited. On one hand, clinicians must consider that periodontal indices are ordinal variables and if the mean difference between both treatment approaches is between 0 and 1, then probably both treatment approaches will receive the same index score since the difference is under one full point. On the other hand, the reduction of PPD, despite being statistically significant, could have a limited clinical relevance since the difference in PPD is less than one millimeter which would be practically negligible in a clinical environment.

The results of the present review must be interpreted with caution due to the high risk of bias of the included studies. Only Chhibber *et al*. (4) conducted a randomized clinical trial with 18 months follow-up that could be considered at low risk of bias. Although they reported better periodontal indices for CA particularly at 9 months follow-up, they could not formally conclude that CA has a positive effect on the periodontal health maintenance. The results from this well-conducted randomized clinical trial are in accordance with the results of the present review since they observe a tendence to maintain better periodontal health during the orthodontic treatment with CA but it is not strong enough to drawn a recommendation. Indeed, the results of the present review differ from the results published by Jiang *et al*. (3), probably due to the methodological differences and the inclusion of recently published studies, such as this randomized clinical trial (4).

Periodontal health is strongly related with the presence of dental plaque and the oral hygiene habit of patients. Therefore, having an adequate plaque control, orthodontic treatment itself will not has any negative effect on periodontal health (2,30) Moreover, studies assessing different oral hygiene procedures reported that electric toothbrushes, interproximal brushes, and fluoride toothpaste have a significant beneficial effect on periodontal health during orthodontic treatment (31-33).

The relationship between periodontal health and orthodontic treatment is widely assessed in the literature. Bollen *et al*. (13) in a systematic review conclude that there is not enough evidence about the effects of orthodontic treatment on periodontal health and clinical attachment level. Nevertheless, Ristic *et al*. (34) found that FA transitionally increase the values of all periodontal indices and stimulate the growth of periodontopathogen bacteria. From a microbiological point of view, FA might be associated with a quantitative increase and qualitative change of periodontopathogen subgingival microbiota (35).

Interestingly, Ristic *et al*. (34) found that the maximum values for PPD and number of periodontopathogen microorganisms were reached 3 months after the placement of orthodontic appliances and decreased 6 months after. This finding is important when interpreting the results of the present review as most orthodontic treatments last more than 12 months and the follow-up of the included studies is limited. In the present review, only 4 studies (4,5,24,25) have a follow up of at least 12 months, hence long-term results should be interpreted with caution.

Taking into account the high prevalence of periodontal diseases and conditions, clinicians should assess the baseline periodontal status and predisposing or precipitating factors for periodontal disease and introduce orthodontic patients in personalized oral health programs (14). Gray and McIntyre (36) conclude that oral health promotion programs for patients undergoing FA orthodontic treatment reduce plaque accumulation and improve gingival health in a short-term. This issue could not be so outstanding in CA treatment since a systematic review found better periodontal health, as well as lower quantity and quality of plaque in patients treated with CA than with FA, probably due to the removal of appliances to have an optimal oral hygiene (1).

To the best of our knowledge, only one study assessed the development of gingival recessions in patients under treatment with CA. Madariaga *et al*. (8) found a significant increase of gingival recessions at 3 months follow-up in patients treated with FA but not with CA. This study reports interesting results in a short-term follow-up but needs to be confirmed by long-term follow-up prospective studies with larger sample. If confirmed, it could be of great interest given that Chambrone and Tatakis (37), in a recent systematic review, concluded that non-treated gingival recessions have a high probability of increasing in the long term, even in patients with good oral hygiene. Clinicians should avoid producing iatrogenic recessions during orthodontic treatments; even mild recessions of 0.5mm.

Various limitations arise from this review that must be considered when interpreting the results. Most of the included studies are observational and there is a limited number of randomized clinical trials published on this topic. We also found high heterogeneity among studies regarding periodontal indices and follow-up intervals assessed. Since non-randomized studies were included in the review, we used the difference between a follow-up visit and the baseline index scores as the outcome, instead of considering only the index score in each follow-up time point, in order to reduce a possible source of bias.

High heterogeneity was detected in all the meta-analysis (I2 >90%). This could be due to different sources of co-variances such as the oral hygiene habit of the enrolled patients and the frequency of oral hygiene instructions given by the clinician. Some studies include all the patients in a strict periodontal maintenance program, while others only provide patients with oral hygiene instructions at the baseline. The age of the participants could be another cofounding factor since we included from teenagers to adult patients. Although the high heterogeneity, we could not perform subgroups meta-regressions due to the limited number studies included and the low methodological quality of the studies, hence, no sensitivity analysis was attempted.

Further studies assessing periodontal health and gingival recession during orthodontic treatment with FA and CA are necessary to drawn concise conclusions on this topic. Especially randomized clinical trials with long term follow-up, adhering to consolidated standards of reporting trials guidelines such as the CONSORT statement (38).

Hence, neither CA nor FA seem to have a substantially impact on periodontal health when appropriate oral health promotion programs are established. Based on the limited evidence, up to the date there is not enough evidence to formally conclude that CA maintain better periodontal health during an orthodontic treatment. There is not enough evidence supporting that FA might produce or increase more gingival recessions compared with CA, hence more studies are necessary.
